# Validation and Discriminant Analysis of the Athens Insomnia Scale in Older Adults

**DOI:** 10.1002/mpr.70017

**Published:** 2025-05-30

**Authors:** Huseyin Elbi, Melike Batum, Ece Ozlem Oztürk, Merve Vatansever Balcan, Ayşin Kisabay Ak, Hikmet Yilmaz, Omer Aydemir

**Affiliations:** ^1^ Department of Family Medicine Faculty of Medicine Manisa Celal Bayar University Manisa Turkey; ^2^ Department of Neurology Faculty of Medicine Manisa Celal Bayar University Manisa Turkey; ^3^ Department of Psychiatry Faculty of Medicine Manisa Celal Bayar University Manisa Turkey; ^4^ Department of Family Medicine Faculty of Medicine Manisa Celal Bayar University Manisa Turkey; ^5^ Department of Neurology Faculty of Medicine Manisa Celal Bayar University Manisa Turkey; ^6^ Department of Psychiatry Faculty of Medicine Manisa Celal Bayar University Manisa Turkey

**Keywords:** athens insomnia scale, insomnia, reliability, validity

## Abstract

**Objective:**

This study aimed to validate and discriminate the “Athens Insomnia Scale” in aged.

**Methods:**

Patients were assessed using the Athens Insomnia Scale (AIS) and the Pittsburg Sleep Quality Index (PSQI). The internal consistency of the AIS was assessed using the Cronbach alpha coefficient. For validity analyses, the scale's structure validity was evaluated using confirmatory factor analysis (CFA). Besides, known group analyses were performed for demographic features such as age and education variables, and diagnostic groups were analyzed.

**Results:**

Cronbach's alpha of AIS was found to be 0.881. In the CFA, the goodness of fit indices of the two‐factor model was found to be significant, which supported the two‐dimension structure as a nocturnal sleep problem and its effects on daytime dysfunction. A high correlation was observed between the AIS and the PSQI. It was determined that the discrimination of the > 65 patients from the < 65 patients with AIS was very high, with the discriminating item “frequent nighttime awakenings” in older adults (Wilks' lambda = 0.874).

**Conclusion:**

The Athens Insomnia Scale is a practical, reliable, and useful tool for evaluating insomnia symptoms in older adults. Its two‐dimensional structure distinguishes nocturnal sleep problems and their effects on daytime functionality.

## 1 Introduction

The rapid aging of the world population has led to increased research on health problems in older adults. Sleep disorders, in particular, are a common problem in this age group. Insomnia is one of the most commonly reported sleep problems in older adults and is considered a disorder determined by both subjective symptoms and diagnostic systems (Cruz et al. 2022; Lavoie et al. 2018). According to current estimates, 30%–48% of older individuals have insomnia symptoms, while 12%–20% are diagnosed with insomnia disorders (Peng et al. 2021; Patel et al. 2018).

Insomnia not only reduces quality of life but is also associated with serious medical health problems such as cardiovascular disease, cognitive decline, and depression. Furthermore, it is critical to better understand and manage this problem due to its potential negative impact on quality of life, such as increased risk of falls in this age group (Medic et al. 2017; Berkley et al. 2020). Various demographic, psychosocial, and clinical factors contribute to the development of insomnia in older adults (Li et al. 2018). Risk factors such as female gender, low socioeconomic status, smoking, alcohol consumption, and decreased physical activity have been consistently identified in the literature. In addition, chronic diseases, depression, obstructive sleep apnea, and restless legs syndrome often co‐occur with insomnia (Peng et al. 2021; Jaussent et al. 2011).

In recent years, there has been a significant increase in studies on the use of insomnia assessment scales in older individuals (Jesudoss et al. 2023, 10). Various factors that increase the risk of insomnia in older individuals have been associated with variables such as gender, socioeconomic status, lack of physical activity, and chronic diseases (Peng et al. 2021; Berkley et al. 2020). In addition, new studies are being conducted to increase the reliability of insomnia measurement tools in individuals over 65. Considering both the Insomnia in the Elderly Scale (IES) validation study and the data from the Athens Insomnia Scale (AIS) scale in Taiwan conducted in older adult outpatients, it is also clear that insomnia scales frequently used in this age group need to be further validated for this cohort (Peng et al. 2021; Pradhan and Saikia 2024). This study aims to evaluate the validation of the Turkish form of the Athens Insomnia Scale and perform discriminant analysis in individuals aged 65 and over.

## 2 Methods

### 2.1 Type of Research and Sample Size

The sample size of this methodological study was determined as 10 times larger for the eight‐item scale and planned as at least 100 patients (Boateng et al. 2018). Patients over 65 who applied to Manisa Celal Bayar University, Hafsa Sultan Hospital, Outpatient Departments of Family Medicine, Neurology (Sleep Polyclinic), and Psychiatry with subjective insomnia complaints and volunteered to participate in the study were included. Insomnia diagnosis was made according to DSM‐5 with a clinical interview. The presence of neurocognitive dysfunction, psychotic disorders, chronic neurodegenerative disease (Alzheimer's disease and Epilepsy), and use of sedative and hypnotic drugs were considered as exclusion criteria. Participants completed the data collection tests themselves under the supervision of the research team. Twenty‐two patients were excluded from the study because they could not complete the procedure, and 15 patients did not want to participate. The study was finalized with a total of 110 patients. All patients underwent a clinical interview according to DSM‐5 and were examined for all relevant symptoms of cognitive dysfunction. Other causes of insomnia (such as OSA, RLS, and RBD) were excluded by questioning the clinical findings of these mentioned diseases (snoring, witnessed apnea, nocturia, mouth disorder, excessive daytime sleepiness, abnormal movements and behaviors during sleep, general restlessness). Cases with three of these symptoms were not included in the study.

### 2.2 Adaptation Process and Initial Findings of the Turkish Scale

In the first phase of the validation study of AIS, validation of the Turkish form of the scale was performed in adult patients with the approval of the local Ethics Committee (Ethics committee date/decision no: 08.12.2021/1079) was completed in December 2022 and was published previously (Elbi et al. 2024). In the data obtained in this study, preliminary statistical data pointed out that the scale could be used more efficiently in individuals aged 65 years and over with insomnia complaints, and the need for further analysis in the relevant population was identified. This study was planned as a follow‐up study and was conducted by the research team of the first study. The data of the patients aged 65 years and over (*n* = 65) in the first study (Elbi et al. 2024) were also used. The data from individuals under 65 in the first study (*n* = 195) were used only to assess discrimination between individuals under and over 65 with AIS. The same data collection tools were used in both studies. As a result, validity and reliability analyses were completed with 110 older adults. In the discriminant analysis, data from 305 participants were used.

### 2.3 Data Collection Tools

In the study, an introductory information form for demographic and clinical features, the Athens Insomnia Scale, and Pittsburg Sleep Quality Index questionnaires were applied to the participants.

#### 2.3.1 Information Form

It consisted of 13 questions to determine the patient's sociodemographic and clinical features, prepared by the researchers following the literature.

#### 2.3.2 Athens Insomnia Scale

The Athens Insomnia Scale (AIS) was developed as a short and easy way to self‐assess the severity of insomnia encountered in various clinical and research settings. Cronbach's alpha coefficient was 0.89, with stable consistency if the item was deleted. In addition, the scale's test‐retest reliability correlation coefficient was 0.89 at 1‐week intervals, and its correlation with the Sleep Problems Scale was 0.90 (Soldatos et al. 2000, 2003).

#### 2.3.3 Pittsburg Sleep Quality Index

The Pittsburg Sleep Quality Index (PSQI) is a scale that evaluates a person's sleep quality and disorders. It was developed by Buysse and colleagues in 1989 (Buysse et al. 1989). The validity and reliability study of the Turkish form was also performed (Agargun et al. 1996). A meta‐analysis evaluating the diagnostic accuracy of screening tools for insomnia reported that the PSQI can be used to screen individuals with insomnia, regardless of the underlying cause (Chiu et al. 2016).

### 2.4 Procedure

#### 2.4.1 Adaptation of Athens Insomnia Scale to Turkish and Content Validity

The translation procedure of the Turkish form AIS was fulfilled and reported in the previous study (12).

### 2.5 Data Analysis

Data analysis was performed using R‐based Jamovi software.

The Cronbach alpha coefficient was calculated to evaluate internal consistency, and > 0,7 was considered significant in reliability analyses. Besides, item‐total score correlation coefficients were obtained. In validity analyses, for structure validity, confirmatory factor analysis was performed. For confirmatory factor analysis, the adequacy of the sample was first examined using Bartlett sphericity test and Keiser‐Meier‐Olkin Analysis. Model fit indices, including the root mean square error of approximation (RMSEA), the comparative fit index (CFI), and the Tucker—Lewis Fit Index, were used. According to previous studies, model fit was accepted as “sufficient” when RMSEA ≤ 0.08 and CFI ≥ 0.90, respectively. Items with factor loadings above 0.4 were taken into consideration. For criterion validity, correlation coefficients between the total score and subscores of AIS and PSQI were calculated for concurrent validity and between its subscales. Independent variables such as gender, marital status, income perception, and education status were analyzed using the Mann‐Whitney U and Kruskal‐Wallis tests in known group validity analyses.

### 2.6 Ethical Aspects

Constantin R. Soldatos was contacted via e‐mail to obtain permission to conduct the scale's Turkish validation study. Since the study was intended to continue with a vulnerable population after the validity and reliability study, a separate approval was obtained from the Manisa Celal Bayar University Health Sciences Ethics Committee (Ethics Committee date/decision no: 20, 12, 2023/2138). The study was conducted in accordance with the principles of the Declaration of Helsinki. Written voluntary consent was obtained from the participants.

## 3 Results

The mean age of the research sample was 71.67 ± 5.68 (between 65 and 97) years; the majority (55.5%) were male and married individuals (80.0%). Of the participants, 12.7% had a history of psychiatric disorders such as depression (n:8, 7.2%) or anxiety disorder (n:6, 5.4%) (Table 1).

**TABLE 1:** Demographic and clinical features of the study group **(*n* = 110)**.

Features	*n*	%
Gender		
Female	49	44.5
Male	61	55.5
Marital status		
Married	88	80.0
Single	22	20.0
Education		
Primary school	93	84.5
High school	17	15.5
Income		
Income is less than expenses	15	13.6
Income is equal to expenses	83	75.5
Income is more than expenses	12	10.9
Living area		
Rural	89	80.9
Urban	21	19.1
Living with		
Spouse and/or child	92	83.6
Alone	18	16.4
Psychiatric illness		
Yes	14	12.7
No	96	87.3

### 3.1 Distribution Characteristics of the Data and Reliability Analysis of AIS

The mean total score of AIS of 110 participants is 8.02 (standard deviation [SD] = 5.35). As in its original structure, the Cronbach alpha coefficient for the total scale was calculated as 0.881. The stability of the Cronbach alpha for each item if deleted was observed (alpha varied between 0.852 and 0.890 if the item was deleted). It was obtained that the item‐total score correlation coefficients of the scale ranged between 0.391 and 0.780. Since all the coefficients for each item were significant, it was decided not to remove any items from the scale (Table 2).

**TABLE 2:** Descriptive statistics of the Athens Insomnia Scale, item‐total correlation coefficients, Cronbach's alpha values ​​of the dimensions, and Cronbach's alpha values ​​when the item was deleted.

AIS items	Scores distribution	Item total correlation	Cronbach's alpha value when the item is removed	Cronbach's alpha value
	Mean ± SD			
AIS total				0.881
AIS1	1.24 ± 0.985	0.505	0.882	
AIS2	1.42 ± 0.850	0.630	0.868	
AIS3	0.82 ± 0.960	0.666	0.864	
AIS4	0.85 ± 0.890	0.731	0.857	
AIS5	1.15 ± 0.907	0.780	0.852	
AIS6	0.83 ± 0.833	0.776	0.854	
AIS7	0.86 ± 0.933	0.724	0.858	
AIS8	0.87 ± 0.879	0.391	0.890	

Abbreviations: AIS, Athens Insomnia Scale; SD, standard deviation.

### 3.2 Validity Analysis (*n* = 110)

Confirmatory factor analysis (CFA) was studied for the entire scale of structure validity. The fit indices for the total scale with a single‐dimensional solution were sufficient for the total scale, although the RMSEA coefficient was > 0,10, indicating poor fit. Therefore, a two‐dimensional model similar to the CFA model with a two‐dimensional solution used in the general population was tested. As a result, similar to the original study of the scale, the first five items evaluating nocturnal sleep problems and the last three items evaluating daytime dysfunction were subjected to CFA; the goodness of fit indexes of the model testing two factors were higher, and the error rate was lower than expected (Table 3).

**TABLE 3:** The results of the confirmatory factor analysis of the Athens Insomnia Scale's goodness of fit values for individuals over 65.

Criteria	AIS (one dimensional produced by CFA)	AIS (two dimensional produced by CFA)
χ2	57.987	30.580
df	20	19
χ2/df	2.899	1.609
Root mean square error of approximation (RMSEA)	0.132	0.075
Comparative fit index (CFI)	0.911	0.973
Incremental fit index (IFI)	0.912	0.973

Abbreviations: AIS, Athens Insomnia Scale; CFA, Confirmatory Factor Analysis.

Table 4 shows the standard factor loadings from the CFA using the two‐factor model. In reliability analysis, for internal consistency, Cronbach's alpha coefficients for the total scale and the AIS subscales were 0.881 (the total scale), 0.850 (factor 1; insomnia symptoms), and 0.791 (factor 2; daytime functioning), respectively.

**TABLE 4:** Factor structures of two‐dimensional AIS for older adults (confirmatory factor analysis).

Item	F1: Nocturnal sleep problem	F2: Daytime dysfunction
1. Sleep induction	0.824	
2. Awakenings during the night	0.693	
3. Final awakening earlier than desired	0.578	
4. Total sleep duration	0.791	
5. Overall quality of sleep	0.791	
6. Sense of well‐being during the day		0.629
7. Functioning (physical and mental) during the day		0.659
8. Sleepiness during the day		0.902

Abbreviation: AIS, Athens Insomnia Scale.

### 3.3 Discriminant Analysis (*n* = 305)

The mean and standard deviation of the total scores of the Athens Insomnia Scale were found to be 9.93 ± 5.46 in young adults (*n* = 205, mean age 40.37 ± 12.68, between 18 and 64) and 8.02 ± 5.35 in older adults (*n* = 110). According to the two‐sample *t*‐test result, older adults' total score was significantly lower (*p* = 0.004). Furthermore, the Cronbach alpha coefficient of the total scale was calculated as 0.870 for young adults and 0.881 for older adults.

In addition, it was determined that the discrimination between the age groups (< 65 years and > 65 years) of the AIS was significant (Wilks' lambda = 0.874, *p* < 0.001), and the discriminating item was found as “Awakenings during the night” with a standardized canonical discriminant function coefficient found to be 0.919. The rate of correctly classified cases according to age groups with AIS was 63%.

### 3.4 Concurrent Validity Analysis (*n* = 110)

The correlation coefficients between the total score and subscale scores of the Athens Insomnia Scale and the total score of the PSQI were between 0.623 and 0.814. All the correlations between the two scales and all their subscales were significant (*p* < 0.01, Table 5).

**TABLE 5:** Inter‐scale correlation coefficients.

Scales	Sub‐dimensions and total athens insomnia scale		
	Nocturnal sleep problem	Daytime dysfunction	AIS total
Pittsburgh Sleep quality index total score	0.814^a^	0.623^a^	0.812^a^

Abbreviation: AIS, Athens Insomnia Scale.

^a^ *p* < 0.001.

### 3.5 Known Groups Validity (*n* = 110)

As a result of the univariate analyses, no significant difference was observed in the correlation analyses concerning variables such as gender, education, or diagnosis of major depressive disorder and/or anxiety disorder in terms of the total score of the scale. It was observed that the Athens Insomnia Scale was not significantly affected by demographic and clinical features (Table 6).

**TABLE 6:** The ability of the Athens Insomnia Scale to detect differences for individuals over 65 years according to basic descriptive characteristics (*n* = 110).

Features	Mean ± SD	Statistical value
Gender		
Female	8.00 ± 5.68	*Z* = −0.178 *p* = 0.859
Male	8.03 ± 5.12	
Marital status		
Married	7.80 ± 5.55	*Z* = −1.194 *p* = 0.232
Single	8.91 ± 4.46	
Education		
Primary school	8.04 ± 5.33	*Z* = −0.249 *p* = 0.804
High school	7.88 ± 5.61	
Income		
Income is less than expenses	9.33 ± 5.51	*F* = 0.396 *p* = 0.674
Income is equal to expenses	7.73 ± 5.50	
Income is more than expenses	8.33 ± 4.03	
Living area		
Rural	7.88 ± 5.39	*Z* = −0.716 *p* = 0.474
Urban	8.62 ± 5.24	
Living with		
Spouse and/or child	7.98 ± 5.46	*Z* = −0.344 *p* = 0.731
Alone	8.22 ± 4.89	
Psychiatric illness		
Yes	9.21 ± 6.29	*Z* = −0.616 *p* = 0.538
No	7.84 ± 5.21	

Abbreviation: **SD,** standard deviation.

## 4 Discussion

Changes in sleep architecture, such as increased sleep fragmentation and decreased non‐REM sleep duration during the night, increase insomnia symptoms with aging (Lavoie et al. 2018; Li et al. 2018). To evaluate sleep disturbances in older age, reliable and valid tools specific to this population are needed. This present study demonstrated that the Athens Insomnia Scale meets these requirements and is an effective tool for assessing insomnia symptoms in older adults.

The findings of this validation study conducted in older adults show that the AIS has a high internal consistency (Cronbach's alpha = 0.881). This result is consistent with the findings of the scale's original validation study (Soldatos et al. 2000) and the adaptation studies conducted in different cultural groups (Gómez‐Benito et al. 2011; Okajima et al. 2013; Enomoto et al. 2018). It was also observed that a higher internal consistency coefficient was obtained when compared with the coefficient found in the validation study conducted in the general population, which is a prominent finding of this study (Elbi et al. 2024). In the item‐total score correlation analysis, statistically significant positive correlation values for every item show that the scale is highly reliable.

The high goodness of fit indices of the two‐factor model of the AIS provides evaluation for both nocturnal sleep symptoms and the effects of these symptoms on daytime dysfunction in older adults. In other validation studies of AIS in older adults, it has also been observed that it has a two‐factor structure, namely nocturnal sleep problems and daytime dysfunction (Okajima et al. 2013; Chiang et al. 2009). When this two‐factor structure was considered, as in the Japanese study, the nocturnal sleep problem subscale had higher fit indices ​​(Iwasa et al. 2018). This result shows that the AIS shows similar results in determining the complaint of nocturnal insomnia caused by physiological changes that occur with aging. However, this study obtained the highest value in the factor analysis for sleepiness during the day. This suggests that the AIS may be an effective assessment tool in evaluating the effects of insomnia on daytime dysfunction in older adults.

The fact that the total scores of the AIS differ between young (< 65 years) and old (> 65 years) adults suggests that the scale may have discriminant validity specific to age groups. This finding is supported by the analysis showing that the scale is more sensitive in evaluating nighttime sleep quality in older individuals than the young adult population, with “frequent nighttime awakenings” discriminating the older adult population from the younger adult population. Although it is stated in the literature that changes in sleep patterns during the aging process increase insomnia symptoms (Vitiello et al. 2022; Gotfried et al. 2024), a study conducted among Americans reported that older adults had fewer sleep problems than younger individuals (Kessler et al. 2012). Therefore, it cannot be stated with certainty that the AIS can provide a more sensitive measurement in this age group. Still, the scale was observed to be useful in evaluating nighttime sleep quality in older individuals.

In the concurrent validity analysis, the fact that AIS has a strong correlation with the PSQI supports the ability of the scale to assess sleep quality. The Pearson correlation coefficient of 0.812 clearly shows the strong relationship between the two scales. The concurrent validity of the Athens Insomnia Scale and the Pittsburgh Sleep Quality Index is consistent with other validation studies supporting the competence of both measurement tools in identifying sleep‐related problems. These findings again confirm the effectiveness of AIS in detecting sleep disorders.

In this study, the fact that demographic variables did not significantly affect the mean total scores of AIS indicates that the scale can be applied to different socioeconomic and demographic groups. In a study evaluating insomnia with the AIS in older adults in Taiwan, it was shown that the scale had a significantly higher discrimination feature in the male gender. Still, education, marital status, and residential status did not affect the total score (Peng et al. 2021). In addition, the fact that the level of education did not affect the scale's total score indicates the AIS's ease of use and comprehensibility. This supports the scale's applicability among individuals with low levels of education.

It is known that insomnia and psychiatric conditions such as depression and anxiety often overlap (Palagini et al. 2024; Fang et al. 2019). At the same time, in the validation study of the Insomnia in the Elderly Scale (IES), it was stated that other insomnia scales were developed mainly with participants consisting of psychiatric patients and that they may be more limited in the evaluation of insomnia in the general population older adult group. In this present validation study, where the rate of individuals with psychiatric disorders was higher than the rate expected in the general population, the fact that the Athens Insomnia Scale can evaluate insomnia independently of psychiatric diseases such as depression and anxiety disorder showed that the scale could be a valid tool in assessing insomnia symptoms independently of the effects of these psychiatric conditions.

### 4.1 Strengths and Limitations

One of the most significant strengths of this study is that it examines the validity and reliability of the Athens Insomnia Scale in older adults in detail. The fact that the study was conducted with a sample covering a wide age range increased the generalizability of the findings. It was observed that the scale's internal consistency was high (Cronbach's alpha = 0.881), and the item‐total score correlations presented a strong structure. In addition, the two‐factor model with high goodness of fit indices indicates that the scale increases its capacity to distinguish between nighttime insomnia symptoms and its effects on daytime functioning. The strong correlation obtained in the concurrent validity analyses with the PSQI revealed the consistency and reliability of the AIS with the most widely used scale. The applicability of the scale, independent of education and socioeconomic variables, is an essential advantage in terms of ease of use.

However, the study has some limitations. The fact that 12.7% of the sample had a psychiatric history necessitates testing the AIS with a broader scope in the general population. The need for more than one‐dimensional model in the factor analysis results indicates that the theoretical basis of the scale needs to be tested further. The fact that the study was conducted only in a local population provides limited information about the scale's validity in different cultural contexts. In addition, the high proportion of male participants may have needed to adequately reflect the scale's performance in women. The lack of comprehensive evaluation of socioeconomic factors also limited the generalizability of the results in different demographic groups.

## 5 Conclusion

The study demonstrates that the Athens Insomnia Scale is a reliable and effective tool for assessing insomnia in older adults. The high internal consistency coefficient and strong concurrent validity analyses support using the scale in this population. In particular, the two‐dimensional structure, which provides the capacity to assess nighttime symptoms and daytime dysfunction separately, makes the AIS an ideal assessment tool for older individuals. However, considering the study's limitations, additional studies are needed to evaluate the performance of the scale in different cultural contexts, larger sample groups, and individuals with psychiatric disorders. In future studies, it will be essential to expand the applicability of the AIS independent of demographic factors and to examine the scale's sensitivity in terms of older adults in more detail.

Since elderly individuals tend to use medical services more frequently, and due to neurodegeneration leading to impaired melatonin production, they experience a decline in sleep quality. As a result, sleep disturbances in geriatric outpatient departments tend to become more common. The Athens Insomnia Scale, which is user‐friendly, simple, and practical for daily use, is highly recommended for screening sleep disturbances in the daily practice of geriatric clinics.

## Author Contributions

**Huseyin Elbi:** conceptualization, investigation, methodology, writing – original draft, writing – review and editing, project administration, validation, formal analysis, data curation. **Melike Batum:** investigation, writing – original draft, data curation, resources, software. **Ece Ozlem Oztürk:** investigation, data curation, resources. **Merve Vatansever Balcan:** investigation, data curation, resources. **Ayşin Kisabay Ak:** conceptualization, investigation, data curation, writing – original draft. **Hikmet Yilmaz:** conceptualization, writing – original draft, writing – review and editing, supervision, visualization. **Omer Aydemir:** conceptualization, writing – original draft, writing – review and editing, supervision, visualization.

## Conflicts of Interest

The authors declare no conflicts of interest.

## Data Availability

The data presented in this article are available upon request. To access the datasets, contact the corresponding author.
